# Case of Extra-Uterine Pregnancy

**Published:** 1804-04-01

**Authors:** T. M. Kelson

**Affiliations:** Seven Oaks, Kent


### Case of Extra-uterine Pregnancy; *communicated by* Mr. T. M. Kelson, *of Seven Oaks, Kent.*

In the month of June, 1801, I was sent for to Mrs. Townsend, the wife of a reputable farmer in an adjoining parish, who was suffering very considerable pain from a partial suppression both of urine and stools. Upon inquiry I found she had sufficient reason to suppose herself about ten weeks gone with child, having missed her courses two successive periods, a very trifling appearance at the end of each month excepted. During this visit I did not think it necessary to examine her, but ordered an opening medicine which afforded her much relief. In the course of a few days she was suffering again in the same way, and was relieved by the same means. Shortly after a total suppression of urine took place, and upon examination I discovered, as I then supposed, that it arose from a retroversion of the uterus, the lower part of the pelvis being completely filled with a hard tumour and the os tincæ not to be found without much difficulty. I relieved her with the common female

 catheter, catheter, to which I was obliged to have recourse for a fortnight longer. At the end of that time the impediment, as I had foretold, was suddenly removed, the uterus taking pretty much its natural situation. From this time she went on in the usual way, gradually increasing in size, and the motion of the child became daily more and more sensibly felt, and as she further advanced it became very strong, but she always told me that it was very different to what she had felt with her former children, that it appeared never to move in front, but on each side and at her back bone; this I little attended to, as I was then quite unsuspicious of her true situation. I had fixed her time for the middle of January 1802. She herself thought that it would be in the beginning of the month, and on the first evening of the month she was in so much pain as to send for me; I found upon going to her that her pains were periodical, and had quite the appearance of beginning labour, however as they were not very quick or pressing, and having another engagement, I left her with the full expectation of being again called in the night; but that was not the case. I learnt the next morning that the same sort of pains continued three or four hours after I left her, that she then went to sleep, and was quite free from them upon waking; the child at this time was in strong motion. From this period I heard no more of her for three weeks, when I was called in great haste, and upon my arrival the pains were much stronger and quicker than before, so much so, that I was induced to remain with her five or six hours. They then having gradually subsided, I went away without examining her. After this I visited her daily; she continued languid and unwell, and on the fourth day she had shiverings, succeeded with some feverish heat. Her breasts began filling with milk, and by no means in a small degree, for on the fifth day they were as painfully distended as one usually meets with in a healthy woman who had been delivered that time. All these symptoms were more than sufficient to make me suspect, what upon examination evidently appeared to be the case, that the fœtus was extra-uterine. Upon the first and every other examination, I found the parts somewhat in confusion, the child plainly to be felt through the vagina, the uterus not enlarged, but forced upwards and forward, the os tincæ quite closed; from this last day of pain, all motion of the child ceased. A month after she became regular in her female health, and has without interruption continued so to this time. From the constant weight of the the tumour it appears to be sunk lower, and I think somewhat lessened in size, but she has still the appearance of a woman far gone with child; there are prominent and pretty sharp points in the tumour, which when pressed cause considerable pain to the intermediate parts, and there appears on the left side to be nothing more intervening than the muscles and integuments. It is difficult to account for its quiet and inoffensive retention. Hitherto Nature has made no efforts to get rid of it. From the pressure on the bladder and the rectum, she has frequent inclinations to make water and go to stool, but the general state of her health is very good. She was thirty-seven years of age when this pregnancy first began, and had not been with child for nearly ten years. Before that period she had five children, and her last labour she describes as a very difficult one. She is naturally a thin, spare chested woman, having scarcely the appearance of breasts when not with child; but what is peculiar, they are now very large, and have always some milk in them. For a considerable time after the death of the child, the milk was continually running out. At the end of two months, I think she would not have made a bad wet nurse.

This Case I meant for publication in the month of April last year, thinking it might be a great length of time, if ever, that I should have any thing further to add, but on the very day I drew it up my patient sent for me. I found her in bed with a very severe attack of influenza, which terminated in a violent cough. This in her situation was particularly painful and troublesome, and seemingly had the effect of altering the position of the tumour, as she again was troubled with suppression of both urine and stools, and was thus tormented, with the addition of sharp throbbing pains in her back, for some weeks. At the same time her general health and appearance were daily getting worse. Menstruation ceased, she had violent night sweats, a total loss of appetite, and other very alarming complaints, when suddenly a large quantity of very putrid slimy matter was discharged per anum; this evacuation, to a certain degree, continued several times a day for a month or more. By the immediate reduction of the tumour and other circumstances, I had no doubt but a communication was formed betwixt it and the rectum. Of this I was shortly fully convinced by the expulsion of the bones of an arm, and since that, at different times, of every bone of the fœtus, excepting those of the head, which I do not despair of adding to my collection. From the time that Nature found out this extraordinary way of getting rid of her burthen, my patient began to improve. She soon became regular, her appetite returned, the cough and night sweats disappeared, and she is now, independent of some local trifling inconvenience, enjoying very good health.

*March* 10th, 1804.

